# Normal-Weight Obesity Is Associated with Poorer Cardiometabolic Profile and Lower Physical Fitness Levels in Children and Adolescents

**DOI:** 10.3390/nu12041171

**Published:** 2020-04-22

**Authors:** Antonio García-Hermoso, Cesar Agostinis-Sobrinho, Gloria Eugenia Camargo-Villalba, Nubia Mercedes González-Jiménez, Mikel Izquierdo, Jorge Enrique Correa-Bautista, Robinson Ramírez-Vélez

**Affiliations:** 1 Department of Health Sciences, Public University of Navarra, Navarrabiomed-IdiSNA, Complejo Hospitalario de Navarra (CHN), 31008 Pamplona, Spain; mikel.izquierdo@gmail.com (M.I.); correab.jorge@gmail.com (J.E.C.-B.); robin640@hotmail.com (R.R.-V.); 2 Laboratorio de Ciencias de la Actividad Física, el Deporte y la Salud, Facultad de Ciencias Médicas, Universidad de Santiago de Chile, USACH, Santiago 7500618, Chile; 3 Faculty of Health and Sciences, Klaipeda University, 92294 Klaipeda, Lithuania; cesaragostinis@hotmail.com; 4 Facultad de Ciencias de la Salud, Universidad de Boyacá, Tunja 150003, Boyacá, Colombia; gloriacamargo@uniboyaca.edu.co (G.E.C.-V.); nubiagonzalez@uniboyaca.edu.co (N.M.G.-J.); 5 CIBER of Frailty and Healthy Aging (CIBERFES), Instituto de Salud Carlos III, 28029 Madrid, Spain

**Keywords:** body composition, normal-weight obesity, body fat, cardiometabolic risk, Latinos

## Abstract

Normal-weight obesity (NWO) syndrome has been shown to be associated with cardiometabolic dysfunction. However, little is known regarding this potential relationship in Latin American children and adolescents. The aim of this study was two-fold: (i) to investigate whether Colombian youth with NWO syndrome have a poorer cardiometabolic profile and physical fitness performance than normal-weight lean (NWL) peers; and (ii) to determine if physical fitness levels are related to prevalence of normal-weight obesity in youth. This was an analytical cross-sectional study of 1919 youths (9–179 years old, 53.0% girls) in the capital area of Colombia. NWO was defined as a body mass index < 25 kg/m^2^ and a validated body fat percentage above the sex-age-specific 90th percentile for Colombian children and adolescents. Body fat was estimated using bioelectrical impedance analysis, cardiorespiratory fitness (CRF) was estimated using the 20-meter shuttle run test, and muscular fitness with the handgrip test. Biochemical profile blood samples were collected for cardiometabolic risk factors. After adjusting for chronological age, pubertal stage, and Mediterranean diet adherence, the NWO group (boys and girls) had significantly higher values for cardiometabolic risk factors, and waist circumference (WC) than the NWL group. The prevalence of NWO was lower in youth classified with healthy CRF (boys, odds ratio (OR) = 0.54, 95% confidence interval (CI) 0.37 to 0.78; girls, OR = 0.35, 95% CI 0.24 to 0.50), *p* < 0.001. Our findings indicate that using only body mass index for the assessment of cardiometabolic risk likely misrepresents true adiposity and suggest the need to include the assessment of body fat in the routine clinical evaluation of individuals during childhood and adolescence.

## 1. Introduction

The World Health Organization (WHO) defines overweight and obesity as abnormal or excessive fat accumulation that may impair health [1]. The WHO has established body mass index (BMI) thresholds to identify overweight and obesity because of their correlations with body fat [2] and cardiometabolic risk factors [3] among youth. While BMI is widely used as a proxy measure of obesity, it does not consider body adiposity, as it does not differentiate lean from fat tissue. Extensive research has shown that excess of adiposity is related to the development of non-communicable diseases such as cardiovascular diseases [1]. Accordingly, youth with normal BMI may have a higher-than-normal percentage of body fat and be at a greater risk of developing non-communicable chronic diseases [4]. Excess body fat despite normal BMI has been termed “normal-weight obesity (NWO) syndrome” [5], and research in adults shows that normal-weight obese individuals are insulin-resistant, hyperinsulinemic, and predisposed to type 2 diabetes mellitus and premature coronary heart disease [6]. 

There has been scarce attention paid to NWO in children and adolescents and, accordingly, little information is available regarding the potential of NWO to impact cardiometabolic health and physical fitness performance in this population. Because cardiorespiratory fitness (CRF) and muscular fitness are related to better cardiometabolic health status among youth [7,8], their levels could be related to NWO. As far as we know, only one study has investigated this issue using data on 182 adolescents in Iceland, finding that NWO was associated with less desirable health behaviors and increased metabolic risk [9]. Considering all this evidence, it seems that further investigations need to be done in young populations, in order to identify and characterize the health risks, complications, and factors associated with NWO. This could offer important insights that would allow the proposal of specific actions into healthcare plans (e.g., periodic assessment of body composition) to limit complications, as NWO youth get older.

Whether healthy levels of physical fitness favor a lower prevalence of NWO in youth is also not known with certainty. Therefore, the aim of the present study was two-fold: (i) to investigate if Colombian youth with NWO have a poorer cardiometabolic profile and physical fitness performance than their NWL peers; and (ii) to determine if physical fitness levels are related to prevalence of NWO.

## 2. Materials and Methods

### 2.1. Design and Study Population

Participants in this study were recruited from the cross-sectional FUPRECOL study [10]. Briefly, the FUPRECOL study was designed to examine the associations between physical fitness levels, body composition, and cardiometabolic risk factors in Colombian youths. Two research groups are involved in the project: (a) Center of Studies in Physical Activity Measurements, School of Medicine and Health Sciences, University of Rosario, and (b) District Education Secretary, Bogotá, DC. All participants were of low–middle socioeconomic status (SES; 1–3 in a scale of 1–6 defined by the Colombian government) and were enrolled in 20 public elementary and high schools (i.e., grades 5–11). The participants answered a questionnaire (paper-and-pencil format) containing information on socioeconomic, demographic, and behavioral variables (i.e., alcohol intake, diet, tobacco) during the 2014–2015 school year. 

The sample consisted of children and adolescents (*n* = 4000 boys and *n* = 4000 girls) aged 9–17.9 years. In a subgroup of 2775 (35%) participants, parameters of biochemical profile were also assessed, and a more comprehensive health and lifestyle assessment was conducted. Of the 2775 participants who took part in the FUPRECOL subgroup study, a total of 1919 (53.0% girls) remained in the present analysis after excluding participants without a 20-meter shuttle run test (20mSRT) score (*n* = 37), body mass index >25 kg/m^2^ (*n* = 455), without a biochemical profile (*n* = 180), and WC (*n* = 184) values. The FUPRECOL study was approved by the Rosario University Institutional Review Board for research on human subjects (Code CEI-ABN026-000262) in accordance with the Declaration of Helsinki (World Medical Association for Human Subjects). 

### 2.2. Anthropometric Measurements 

In order to standardize the evaluation process and to minimize inter-observer variability, six theoretical and practical sessions were run by the researchers to train the staff in all procedures of the study. Body weight and height were measured in underwear and without shoes using an electronic scale (with an accuracy of 0.1 kg) (BF-689 Children’s Body Fat Monitor - Tanita, Tokyo, Japan) and a mechanical stadiometer platform (with an accuracy of 0.1 cm) (Seca 217–213; Seca, Hamburg, Germany), respectively. BMI was also calculated as body weight in kilograms divided by height in meters squared (kg/m^2^). WC was measured to the nearest 0.1 cm at the level of the umbilicus, using a tape measure (W606PM; Lufkin, Parsippany, NJ) and the average of the three measurements was used in the analyses. Intra-observer and inter-observer technical error of measurement (TEM) for WC was (R = 0.979) and (TEM = 1.32 %), respectively. Body fat was assessed using a bioelectrical impedance analysis (Tanita BC 420 MA/SC-331S®; Tokyo, Japan) as described previously [11]. The validity and reliability of these measures have been previously determined [11]. Finally, NWO was defined as a BMI <25 kg/m^2^ and a percentage of body fat over the sex-age-specific 90th percentile for Colombian children and adolescents (boys > 23.4%–28.3% and girls > 31.0%–34.1%) [12]. 

### 2.3. Cardiorespiratory Fitness and Handgrip Strength Parameters 

We assessed CRF by the 20-meter shuttle run test (20mSRT), as described in [13]. Peak oxygen consumption (VO_2_ peak, mL·kg^−1^·min^−1^) was estimated by Barnett et al.’s [14] equation, which has the highest discriminatory accuracy for identifying unhealthy and healthy cardiometabolic risk in Colombian children and adolescents for sex and age (9–17.9 years) [15]. 

Upper muscular strength was assessed using the maximum handgrip strength test with a digital dynamometer (T.K.K. 5401, Grip-D Smedley; Takei, Japan). The final score (kg) was calculated as the average of the scores for both hands. Handgrip strength was normalized as handgrip strength (in kg)/body weight (in kg). Children and adolescents were classified as healthy or unhealthy based on the sex and age cut-off points established previously [16].

### 2.4. Cardiometabolic Risk Factors 

Cardiocheck® equipment (Mexglobal SA, Parsippany, NJ) was used to determine concentrations of glucose, total cholesterol (TC), triglycerides, and high-density lipoprotein cholesterol (HDL-C) levels in capillary blood samples after overnight fasting. Low-density lipoprotein cholesterol (LDL-C) was measured using Friedewald’s formula if TG values were ≤400 mg/dL. All assays were performed in duplicate.

Resting blood pressure was measured with an automated blood pressure monitor (Omrom® HEM 705 CP; Omron Healthcare, Kyoto, Japan). The average of two measurements was recorded with the youth seated in a quiet room.

A cardiometabolic risk score was created from the sum of the z-scores values of systolic blood pressure, serum triglycerides, WC, HDL-C (multiplied by −1), and fasting glucose z-score [17] calculated as follows: (value–mean)/standard deviation (SD), separately for boys and girls, and for each 1-year age-group. A higher cardiometabolic risk z-score is indicative of an unhealthier risk profile [15,16].

### 2.5. Mediterranean Diet Adherence

In order to determine the degree of adherence to the Mediterranean diet, the KIDMED questionnaire was used [18]. This tool has high consistency (α = 0.79) [19] and comprises 16 questions with an affirmative (twelve items) or negative (four items) response. The sum of all values from the administered questionnaire was categorized into two levels: (1) 0–7, poor/average adherence and (2) 8–12, good adherence [18]. 

### 2.6. Pubertal Stage

Sexual maturation was classified based on Tanner and Whitehouse staging of secondary sex characteristics [20] (breast and pubic hair development for girls, genital and pubic hair development for boys; ranging from stage I to V). We used three stages: pre-pubertal stage, pubertal stage, and late/post-pubertal stage.

### 2.7. Statistical Analysis

Sample characteristics were presented as mean and standard deviation (SD) for continuous variables, and frequencies and percentages were calculated for categorical variables. Data normality was verified by both Kolmogorov–Smirnov and graphical methods (normal probability plots).

Differences in mean values of cardiometabolic and physical fitness parameters according to group (i.e., NWL and NWO) were tested by the Mann–Whitney non-parametric test, since all dependent variables were skewed distribution. Differences in categorical variables were analyzed by using a chi-square test. Analysis of covariance (ANCOVA) was also used to compare means of each cardiometabolic and physical fitness parameter according to group (i.e., NWL and NWO) and adjusted for age, BMI, pubertal stage, and Mediterranean diet adherence (all dependent variables were log-transformed before inclusion in the models).

Finally, logistic regression models were employed to determine the odds of being classified as NWO according to CRF categories using unhealthy CRF as a reference after adjusting for chronological age, pubertal stage, and Mediterranean diet adherence. Alpha was set at 0.05. All statistical analyses were performed using SPSS-PC statistical software (version 23.0, SPSS, Inc.). 

## 3. Results

The characteristics of the study group are shown in Table 1. The number of normal-weight youths included in the study was 1919 (53.0% girls), of which 46.0% were classified as NWO (47.0% girls). The NWO group was significantly older than the NWL group (*p* < 0.001). Regarding anthropometric parameters, the NWO group had significantly higher values for body weight, height, BMI, and body fat (all *p* < 0.001) than the NWL group. 

**Figure 1:**
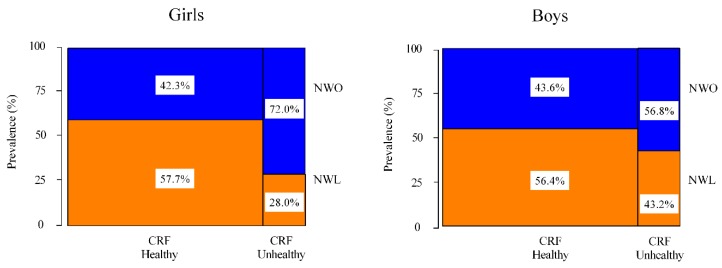
Mosaic plot depicting the frequency of participants (%) in each obesity group (normal-weight lean (NWL), normal-weight obesity (NWO)), cardiorespiratory fitness (CRF) categories (healthy, unhealthy) by sex. NWO was defined as a BMI < 25 kg/m^2^ and a percentage of body fat over the sex-age-specific 90th percentile for Colombian children and adolescents (boys > 23.4%–28.3% and girls > 31.0%–34.1%) [12]. CRF were grouped into two groups (i.e., healthy and unhealthy) according to previously suggested cut-off points associated with high cardiovascular disease risk in Colombian children and adolescents [15]. These cut-off points were defined as < 47.9 mL·kg^−1^·min^−1^ in boys and < 34.4 mL·kg^−1^·min^−1^ in girls aged 9–12.9 years, and < 48.0 mL·kg−1·min^−1^ in boys and < 33.8 mL·kg^−1^·min^−1^ in girls aged 13–17.9 years, respectively.

To illustrate the degree to which both concepts (fitness vs. fatness) overlap, we reported the prevalence of each obesity group (NWL vs. NWO) according to the CRF groups (healthy and unhealthy), by sex (Figure 1). In girls, the category containing the unhealthy CRF plus NWO group was 72.0% vs. 42.3% in the CRF healthy group (*p* < 0.001). In boys, the proportions of subjects with unhealthy CRF plus NWO group were 56.8%, and 43.6% in the CRF healthy group. These findings indicate that those subjects positioned in the healthy CRF and the NWL group present better health status than the NWO group, within unhealthy CRF values, *p* < 0.001.

Table 2 shows differences in cardiometabolic and physical fitness parameters between NWL and NWO youth, with the latter group (boys and girls) having significantly higher values for WC, triglycerides, systolic blood pressure, cardiometabolic risk score, and CRF than the NWL group, and significantly lower values for in HDL-C. After adjusting for covariates, significant results were reduced and even disappeared for HDL-C and systolic blood pressure.

**Figure 2:**
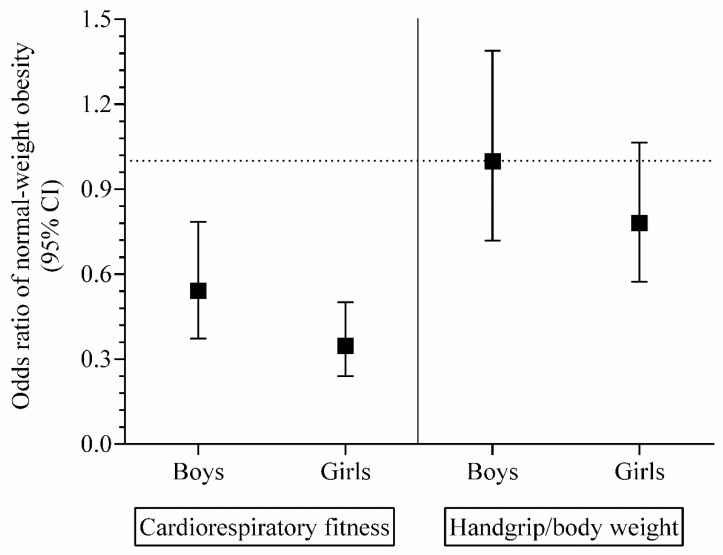
Odds ratios (ORs) to be classified as normal-weight obese according to fitness categories. Reference (OR = 1.0), unhealthy physical fitness level [15] (CRF < 47.9 mL·kg^−1^·min^−1^ in boys and < 34.4 mL·kg^−1^·min^−1^ in girls aged 9–12.9 years, and < 48.0 mL·kg^−1^·min^−1^ in boys and < 33.8 mL·kg^−1^·min^−1^ in girls aged 13–17.9 years, respectively) and unhealthy handgrip/body weight levels [16] (< 0.359 and < 0.376 in girls and boys aged 9–12.9 years, respectively. In adolescents (13–17.9 years), these points were < 0.440 and < 0.447 in girls and boys, respectively).

Finally, the adjusted odds ratios (ORs) to be classified as NWO according to physical fitness parameters (CRF and handgrip strength/body weight) categories are shown in Figure 2. For both sexes, the prevalence of NWO was lower in youths classified with healthy CRF levels (boys, OR = 0.54, 95% CI 0.37 to 0.78; girls, OR = 0.35, 95% CI 0.24 to 0.50) (*p* < 0.001) compared to unhealthy peers.

## 4. Discussion

Our findings reveal that NWO youth have a significantly poorer cardiometabolic profile and level of physical fitness than their NWL counterparts. A healthy level of CRF seems to be related to a lower prevalence of NWO. Our results therefore indicate that using only BMI seems insufficient for the determination of cardiometabolic risk and suggest the need to include the assessment of body fat during childhood and adolescence. Thus, body fat measurement in normal-weight youths will provide additional information and allow us to determine and reduce their possible cardiometabolic risk.

Several recent studies have uncovered metabolic dysregulation in people classified with NWO [6], even among adolescents [9]. Confirming these results, our study suggests that NWO boys and girls show significantly higher levels for WC, triglycerides, systolic blood pressure, and cardiometabolic risk score than their NWL peers, and a lower level of HDL-C. On the basis of published studies, we can speculate that youth with NWO show altered levels of proinflammatory cytokines, which may contribute to an increased risk of developing cardiometabolic disorders [6]. Similarly, Olafsdottir et al. [9] reported that metabolic risk was considerably higher among NWO adolescents than their NWL peers, with high WC being more prevalent in the former. This is consistent with the observation in children and adolescents that subcutaneous adiposity at the waist is a strong predictor of cardiometabolic risk [21]. Taken together, these results suggest the need to establish strategies to screen for increased body fat as early as possible, because cardiometabolic risk parameters track from childhood to adulthood [22].

The benefits of health-related physical fitness during childhood are widely recognized and span many physical and mental health domains [7,8]. Specifically, a low level of muscular fitness is recognized as a marker of poor cardiometabolic and adipose tissue function during childhood and adolescence, and also in later life [8]. Moreover, higher CRF during youth is associated with lower risk of future overweight, obesity, and metabolic syndrome [7]. It is therefore not surprising that the NWL group in the present study showed higher cardiorespiratory and muscular fitness levels than NWO peers. Olafsdottir et al. [9] also reported similar results among adolescents from Iceland, finding that NWO was associated with lower CRF assessed by maximal oxygen uptake during a treadmill test. Consistent with this view, our study reveals that a healthy CRF level, determined with Colombian cut-offs, is related to a lower prevalence of NWO. Our present findings confirm the results of a previous systematic review suggesting that higher CRF in childhood and adolescence is associated with lower BMI and body fatness [7]. In a debate article [23], Carson [24] proposed a possible mechanism to link the development of CRF with the individual trajectories of adiposity and growth; the author suggested that vigorous exercise during the growing years, the type of physical activity that is strongly linked to CRF improvement, promotes the differentiation of stem cells into bone and muscle rather than into fat cells.

Interestingly, the differences between NWO and NWL, particularly among girls, were attenuated and no longer significant for HDL-C and blood pressure upon adjustment for BMI, indicating that NWO status may specifically affect CRF beyond BMI (Table 2). Importantly, although the current study focused on the NWO in children and adolescents, this may not necessarily mean that the established health risks of HDL-C and blood pressure can be neglected. Therefore, the present analysis should be reproduced in a larger sample to validate whether in fact the paths are actually similar in both genders [25]. For this reason, given the potential influence of NWO on physical fitness, the findings of our study highlight the importance of considering body composition and CRF levels analysis [26] rather than only measuring BMI in future preventive studies and practices in a school setting. 

Our study has several limitations. First, the cross-sectional design prevents us from establishing a causal relationship. Second, we predicted VO_2_ peak from a single field test, and standard testing methods with directly measured oxygen consumption are required to confirm our results. Thus, the current results should be interpreted with caution, since no direct measure of absolute VO_2_ peak was available in our study. Third, the study was performed only in Bogotá (Colombia) and therefore the relationships found in the present study need to be confirmed using a representative sample. Despite these limitations, the main strength of our study is that, to our knowledge, this is the largest research on the relationship between NWO and cardiometabolic risk in a population of Latin American children and adolescents. These data from adolescents aged 9–17.9 years complement the study published by Correa-Rodríguez et al. [25] on the use of the NWO in addition to body adiposity parameters in practice by general practitioners, teachers, and coaches in a school setting. Furthermore, highly standardized procedures were developed within the FUPRECOL study to avoid measurement bias [11]. Additionally, statistical models were adjusted for several variables, including age, pubertal stage, and Mediterranean diet adherence.

## 5. Conclusions

The present findings suggest associations between NWO and cardiometabolic and physical fitness parameters in Colombian youth with BMI within the normal range. Studies in child populations are important because if NWO is associated with metabolic imbalances at an early age, clinical evaluation should change and preventive public policy actions should be redrawn and begin earlier, to limit complications as NWO youth get older. Overall, these results indicate the importance of incorporating into routine clinical evaluation or even at school, other simple low-cost measures like skinfold thickness or bioelectrical impedance to evaluate excess body fat among schoolchildren. Future prospective studies on the current topic are therefore recommended.

## Figures and Tables

**Table 1:** Characteristics of the study sample of normal-weight lean (NWL) and normal-weight obesity (NWO) participants.

Characteristics	NWL (*n* = 1030)	NWO (*n* = 889)	*p*
Age, years	12.82 ± 2.11	13.86 ± 2.21	<0.001
Girls, %	53.0	47.0	0.264
Anthropometric parameters			
Body weight, kg	41.09 ± 9.32	47.79 ± 9.66	<0.001
Height, m	1.50 ± 0.12	1.55 ± 1.12	<0.001
Body mass index, kg/m^2^	17.86 ± 1.85	19.67 ± 1.91	<0.001
Body fat, kg	7.24 ± 3.09	9.89 ± 3.56	<0.001
Pubertal stage, %			
Pre-pubertal stage	6.7	4.7	<0.001
Pubertal stage	57.2	43.6	
Late/post-pubertal stage	36.1	51.7	
Mediterranean diet adherence			
KIDMED index score, points	4.0 ± 1.7	3.4 ± 1.6	0.001
KIDMED index score (poor/average/good), %	53.7/36.5/9.9	49.0/41.0/10.0	0.099

Results are shown as mean and standard deviation (SD) for continuous variables, and percentages (%) for categorical variables.

**Table 2:** Differences in cardiometabolic and physical fitness parameters between normal-weight lean (NWL) and normal-weight obesity (NWO) youth.

**Sex**	**Groups**		***p* Value**	*p* Value *
**Boys (*n* = 902)**	**NWL (*n* = 496)**	**NWO (*n* = 406)**		
Cardiometabolic parameters				
Waist circumference, cm	61.78 ± 5.12	65.85 ± 6.13	<0.001	0.042
Triglycerides, mg/dL	76.72 ± 30.8	85.32 ± 38.13	<0.001	0.017
HDL-C, mg/dL	49.60 ± 12.99	45.72 ± 11.75	<0.001	0.317
Fasting glucose, mg/dL	83.36 ± 14.95	81.91 ± 17.04	0.122	0.472
Systolic blood pressure, mm Hg	111.44 ± 14.98	1114.60 ± 13.34	0.001	0.994
Cardiometabolic risk score	−0.97 ± 2.10	−0.15 ± 2.31	<0.001	0.019
Physical fitness parameters				
Cardiorespiratory fitness, mL·kg^−1^·min^−1^	51.36 ± 2.97	50.67 ± 3.19	0.001	0.009
Handgrip strength/body weight	0.50 ± 0.09	0.51 ± 0.11	0.785	<0.001
**Girls (*n* = 1017)**	**NWL (*n* = 539)**	**NWO (*n* = 478)**	***p* Value**	
Cardiometabolic parameters				
Waist circumference, cm	59.12 ± 5.07	63.94 ± 5.89	<0.001	<0.001
Triglycerides, mg/dL	87.16 ± 36.61	97.86 ± 62.59	<0.001	0.065
HDL-C, mg/dL	49.63 ± 12.51	47.15 ± 12.21	<0.001	0.484
Fasting glucose, mg/dL	81.96 ± 15.16	80.47 ± 16.86	0.055	0.051
Systolic blood pressure, mm Hg	107.81 ± 12.31	110.58 ± 12.36	<0.001	0.268
Cardiometabolic risk score	−1.08 ± 2.31	−0.12 ± 2.38	<0.001	0.044
Physical fitness parameters				
Cardiorespiratory fitness, mL·kg^−1^·min^−1^	36.86 ± 2.33	35.56 ± 2.37	<0.001	0.002
Handgrip strength/body weight	0.43 ± 0.07	0.40 ± 0.06	<0.001	<0.001

Results are shown as mean and standard deviation (SD) for continuous variables, and percentages (%) for categorical variables. HDL-C, high-density lipoprotein cholesterol. CRF was calculated with the Barnett et al. [14] equation for each participant as follows = 25.8 × (6.6 × (G × (0.2 × (BM + 3.2 × S), where G is gender (male = 0, female = 1), BM is body mass (kg), and S is final speed. * Analyses were fully adjusted for age, BMI, pubertal stages and Mediterranean diet adherence.
